# Intravitreal aflibercept for active polypoidal choroidal vasculopathy without active polyps

**DOI:** 10.1038/s41598-018-37523-5

**Published:** 2019-02-06

**Authors:** Sang Eun Lee, Jun Won Jang, Se Woong Kang, Kyu Hyung Park, Dong Won Lee, Jae Hui Kim, KunHo Bae

**Affiliations:** 1 Department of Ophthalmology, Samsung Medical Center, Sungkyunkwan University School of Medicine, Seoul, Korea; 2 Department of Ophthalmology, Seoul National University College of Medicine, Seoul National University Bundang Hospital, Seongnam, Republic of Korea; 3 Department of Ophthalmology, Kim’s Eye Hospital, Konyang University College of Medicine, Seoul, Republic of Korea

## Abstract

The purpose of this study was to evaluate the efficacy of intravitreal aflibercept for active polypoidal choroidal vasculopathy (PCV) without active polyps and to identify prognostic factors. We enrolled 40 eyes from 40 patients who manifested PCV with exudation but without active polyps after prior treatment with photodynamic therapy (PDT) and/or anti-vascular endothelial growth factor (VEGF) other than aflibercept. Participants were initially given three consecutive intravitreal injections of aflibercept at 1-month intervals, followed by injections every 2 months in the maintenance phase. Spectral-domain optical coherence tomographic and indocyanine green angiographic features were assessed to determine associations between anatomical parameters and visual outcomes 14 months later. Mean visual acuity improved from 61.5 ± 11.1 letters at baseline to 68.1 ± 13.6 letters at 14 months (*P* = 0.001). Better vision and a smaller branching vascular network at baseline and 1 month after three monthly injections (visit 4) were associated with better final vision (*P* < 0.001). The presence of an inner retinal cyst at visit 4 was significantly related to worse final vision (*P* = 0.011). Intravitreal aflibercept improved the visual and anatomical outcomes of PCV with exudation from BVN after pre-treatment with PDT and/or anti-VEGF other than aflibercept. Better vision, smaller lesion size, and absence of an inner retinal cyst after induction therapy may predict better visual outcome.

## Introduction

Polypoidal choroidal vasculopathy (PCV) is characterized by the occurrence of polypoidal lesions or “polyps” that frequently progress to exudative and hemorrhagic complications in the subretinal or sub-pigment epithelial space. Although the etiology of PCV is still unclear, it has been revealed that PCV is a variant of type I neovascularization, based on OCT images through the PCV complex as imaged on ICG angiography^1–4^.

Anti–vascular endothelial growth factor (VEGF) therapy and photodynamic therapy (PDT) with verteporfin are the current mainstays of treatment for PCV^5,6^. The primary mechanism of action of PDT is eliminating polyps. Thus, PDT is generally performed when active polyps are noted on ICGA. In contrast, the role of anti-VEGF is to resolve exudation by reducing the activity of the entire neovascular lesion, including the branching vascular network (BVN) as well as polyps.

Aflibercept (Eylea, Regeneron, Tarrytown, NY) is a soluble decoy receptor fusion protein that binds to all isoforms of VEGF-A and VEGF-B as well as placental growth factor^7,8^. Intravitreal aflibercept injections in treatment-naive eyes with PCV improved visual acuity and macular morphology^9^. In PLANET study, aflibercept monotherapy without PDT resulted in comparable visual outcomes to combination therapy with aflibercept and deferred PDT^2^. However, as fewer than 15% of the subjects of the study met the criteria for a suboptimal response to receive PDT, the potential benefit of adding PDT cannot be determined.

Many patients have recurrent or refractory exudation but no active polyps because of previous treatment. Thus, how to manage cases of PCV with exudation but without polyps is of real-world importance. Saito *et al*.^10^ reported that ranibizumab was efficacious in patients with PCV with recurrent or residual exudation from BVN after previous PDT. However, the efficacy of aflibercept in recurrent or refractory cases has not been determined. Furthermore, it is not clear if anti-VEGF therapy is effective in cases without active polyps.

Our aims in this study were therefore to evaluate the efficacy of intravitreal aflibercept for PCV with exudation but without active polyps in eyes pre-treated with PDT or anti-VEGF other than aflibercept, and to identify factors related to visual prognosis.

## Results

### Demographic features of the study eyes

A total of 40 eyes from 40 patients were enrolled. All enrolled patients had completed 14 months of follow-up. Mean age (±standard deviation (SD)) was 69.5 ± 8.6 years (range, 53 to 85); 25 patients (62.5%) were male and 15 (37.5%) were female. Twenty-five patients (62.5%) had a history of PDT. Table 1 summarizes the baseline characteristics of the 40 eyes before initiation of aflibercept injections.

**Table 1:** Baseline characteristics.

Baseline characteristic	
Mean age ± SD (years)	69.5 ± 8.6
Gender, n (%)	
Male	25 (62.5)
Female	15 (37.5)
Mean BCVA ± SD (letters)	61.5 ± 11.1
Treatment history	
PDT	25 eyes
Anti-VEGF	40 eyes
Bevacizumab + Ranibizumab	25 eyes
Ranibizumab only	12 eyes
Bevacizumab only	3 eyes
OCT findings	
Mean CMT ± SD (μm)	351.0 ± 94.5
Presence of SRF, n (%)	38 (95.0)
Presence of IRC, n (%)	9 (22.5)
Presence of PED, n (%)	8 (22.0)
Presence of SRH, n (%)	1 (2.5)
Presence of SHRM, n (%)	21 (52.5)
ICGA findings	
Presence of LGH, n (%)	40 (100.0)
Mean LGH area ± SD (OD size)	1.43 ± 0.71
Mean BVN area ± SD (OD size)	0.96 ± 0.56
No. of inactive polyp(s)	0.85 ± 0.98
No. of punctate hyperfluorescence spots in ICGA	
0–2	22 (55.0)
3–10	5 (12.5)
10–20	3 (7.5)
20–40	7 (17.5)
>40	3 (7.5)

SD = standard deviation; BCVA = best-corrected visual acuity; PDT = photodynamic therapy; VEGF = vascular endothelial growth factor; OCT = optical coherence tomography; CMT = central macular thickness; SRF = subretinal fluid; IRC = inner retinal cyst; PED = pigment epithelium detachment; SRH = subretinal hemorrhage; SHRM = subretinal hyperreflective materials; ICGA = indocyanine green angiography; LGH = late geographic hyperfluorescence; BVN = branching vascular network; OD = optic disc.

### Visual outcomes

**Figure 1:**
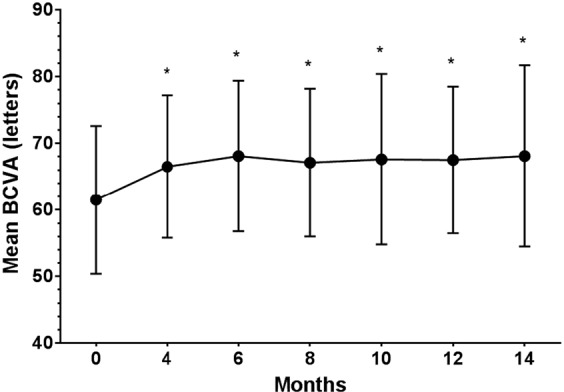
Changes in mean best-corrected visual acuity (BCVA) from baseline. Mean BCVA improved significantly from baseline and was maintained for at least 14 months. *P value < 0.01 compared with baseline (Wilcoxon signed rank test).

Mean best corrected visual acuity (BCVA) using Early Treatment Diabetic Retinopathy Study (ETDRS) charts improved from 61.5 ± 11.1 letters (range, 36 to 77 letters) at baseline to 68.1 ± 13.6 letters (range, 32 to 90 letters) at 14 months (*P* = 0.001). Mean gain of BCVA from baseline to the final visit was 6.6 ± 9.5 letters (range, −25 to 22 letters). Thirty of 40 patients gained BCVA (minimum of 5 letters), five patients lost BCVA, and five patients had the same BCVA as at their baseline visit. Changes in BCVA are shown in Fig. 1.

Better BCVA at baseline and at 1 month after three monthly injections (visit 4) was significantly associated with a better final BCVA (*P* < 0.001 and *P* < 0.001, respectively). The size of the BVN at baseline and at visit 4 was inversely related to final ETDRS letter score (*P* = 0.037 and *P* = 0.031, respectively). The presence of an inner retinal cyst at visit 4 was also significantly related to a worse final BCVA (*P* = 0.011). There were no differences in visual acuity outcomes between patients with and without a history of PDT. Table 3 shows the correlation between various parameters and visual and anatomical outcomes at baseline, 4 months, and 14 months.

### Anatomic outcomes

**Figure 2:**
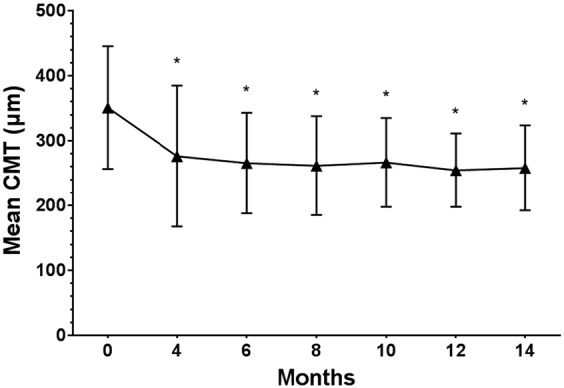
Changes in mean central macular thickness (CMT) from baseline. Mean CMT improved significantly from baseline and was maintained for at least 14 months. *P value < 0.01 compared with baseline (Wilcoxon signed rank test).

Mean central macular thickness (CMT) decreased from 351.0 ± 94.5 μm (range, 200 to 648 μm) at baseline to 258.1 ± 65.7 μm (range, 162 to 430 μm) at 14 months (*P* < 0.001). Mean CMT for each visit is shown in Fig. 2. Anatomic outcomes based on spectral-domain optical coherence tomography (SD-OCT) images at baseline, visit 4, and the final visit are shown in Table 2. The presence of subretinal hyperreflective materials at baseline was significantly related to a thin final CMT (*P* = 0.015), although it was not related to baseline CMT (*P* = 0.538). The less the macular edema at visit 4 was, the less the macular edema at the final visit was (*P* = 0.004). There was no new occurrence of significant subretinal hemorrhage during the follow-up visits.

**Table 2:** Morphologic findings at baseline, 4 months, and 14 months.

SD-OCT findings	Baseline	4 months	14 months
Mean CMT ± SD (μm)	351.0 ± 94.5	276.3 ± 108.7	258.1 ± 65.7
Presence of SRF, n (%)	38 (95.0)	13 (32.5)	15 (37.5)
Presence of IRC, n (%)	9 (22.5)	3 (7.5)	4 (10.0)
Presence of PED, n (%)	8 (22.0)	5 (12.5)	4 (10.0)
Presence of SRH, n (%)	1 (2.5)	1 (2.5)	0 (0.0)
Presence of SHRM, n (%)	21 (52.5)	14 (35.0)	15 (37.5)

SD-OCT = spectral-domain optical coherence tomography; SD = standard deviation; CMT = central macular thickness; SRF = subretinal fluid; IRC = inner retinal cyst; PED = pigment epithelium detachment; SRH = subretinal hemorrhage; SHRM = subretinal hyperreflective materials.

At the final visit, subretinal fluid was more frequently detected in patients who had subretinal fluid at visit 4 (*P* = 0.007). Inner retinal cysts were more frequently detected at the final visit when the patients had an inner retinal cyst at baseline (*P* = 0.029), a larger late geographic hyperfluorescence (LGH) size at visit 4 (*P* = 0.047), or a larger BVN size at baseline or visit 4 (*P* = 0.040 and *P* = 0.033, respectively). Pigment epithelium detachment was more frequently detected at the final visit when the patient manifested with detachment at baseline or visit 4 (*P* = 0.020 and *P* = 0.004, respectively). There were no differences in anatomic outcomes between patients with and without a history of PDT. One patient developed exudation with reactivation of polyps after six aflibercept injections and received PDT as the rescue treatment. Table 3 shows the analysis results for the parameters at baseline and visit 4.

**Table 3:** Significance (*P* values) of association between various parameters and visual and anatomic outcomes at baseline, 4 months, and 14 months.

	Final visual and anatomic outcomes					
	BCVA	CMT	Presence of SRF	Presence of IRC	Presence of PED	Presence of SHRM
Baseline parameters						
BCVA	**<0.001**	0.466	0.509	0.128	0.348	0.794
CMT	0.902	0.253	0.311	0.770	0.992	0.214
Presence of SRF	0.374	0.337	0.971	0.976	0.976	0.711
Presence of IRC	0.205	0.901	0.292	**0.029**	0.966	0.092
Presence of PED	0.810	0.469	1.000	0.793	**0.020**	0.115
Presence of SRH	*0.015	0.917	0.980	0.983	0.983	0.978
Presence of SHRM	0.621	**0.015**	0.224	0.945	0.272	0.169
Number of inactive polyps	0.396	0.413	0.448	0.391	0.177	0.277
BVN size (OD size)	**0.037**	0.850	0.902	**0.040**	0.734	0.496
LGH size (OD size)	0.603	0.544	0.688	0.060	0.365	0.912
Number of punctate hyperfluorescent spots	0.294	0.421	0.456	0.954	0.697	0.337
Parameters at visit 4						
BCVA	**<0.001**	0.580	0.581	0.265	0.917	0.235
CMT	0.160	**0.004**	0.729	0.766	0.501	**0.046**
Presence of SRF	0.576	0.120	**0.007**	0.737	0.090	**0.007**
Presence of IRC	**0.011**	0.132	0.962	0.985	0.204	0.877
Presence of PED	0.190	0.555	0.902	0.440	**0.004**	0.281
Presence of SRH	*0.015	0.917	0.980	0.983	0.983	0.978
Presence of SHRM	0.667	0.069	0.609	0.661	0.513	**<0.001**
Number of active polyps	0.245	0.396	0.978	0.992	0.992	0.978
Number of inactive polyps	0.656	0.393	0.018	0.073	0.252	0.499
BVN size (OD size)	**0.031**	0.657	0.733	**0.033**	0.875	0.573
LGH size (OD size)	0.810	0.328	0.439	**0.047**	0.463	0.857
Number of punctate hyperfluorescent spots	0.294	0.421	0.456	0.954	0.697	0.337

BCVA = best-corrected visual acuity; CMT = central macular thickness; SRF = subretinal fluid; IRC = inner retinal cyst; PED = pigment epithelium detachment; SHRM = subretinal hyperreflective materials; BVN = branching vascular network; OD = optic disc; LGH = late geographic hyperfluorescence; visit 4 = one month after 3 monthly injections.

^*^It is difficult to give statistical meaning because there was only 1 patient having SRH.

**Figure 3:**
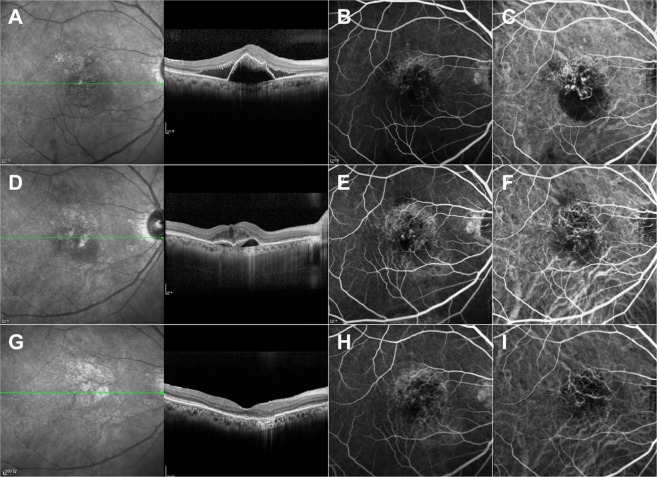
Representative study case. Optical coherence tomography (OCT), early phase of fluorescein angiography, and indocyanine green angiography images of a 72-year-old man diagnosed with polypoidal choroidal vasculopathy. Before baseline (**A–C**), subretinal fluid and pigment epithelium detachment (PED) were present with a branching vascular network and active polyps. The patient was treated twice with photodynamic treatments, four times with ranibizumab injections, and six times with bevacizumab injections. No fluid on OCT was observed even without treatment. Six months after baseline (**D–F**), an inner retinal cyst and PED with a branching vascular network developed, but no active polyps. Early Treatment Diabetic Retinopathy Study (ETDRS) letter score was 71. At 14 months of treatment with a bimonthly fixed dosing regimen of aflibercept injections after three initial monthly injections (**G–I**), the inner retinal cyst and PED had resolved, and his vision improved to an ETDRS letter score of 80.

A representative case is shown in Fig. 3. A 72-year-old man with exudative PCV without polyps received aflibercept treatment. Subretinal fluid resolved completely and the size of the BVN had decreased at 14 months.

### Adverse events

Adverse events were assessed in all patients, and those that occurred during the 14 months of the study are shown in Table 4. There were no major systemic adverse events during the study. Sterile endophthalmitis was noted in one case (2.5%), and was controlled with topical steroids and cycloplegics.

**Table 4:** Adverse events.

Adverse events	n (%)
Ocular	
Floaters	3 (7.5)
Sterile endophthalmitis	1 (2.5)
Pruritus eyelid	1 (2.5)
Dry-eye syndrome	2 (5.0)
Non-ocular	
Right lower leg numbness	1 (2.5)
Cervicalgia	1 (2.5)
Ileus	1 (2.5)

## Discussion

This prospective, single-arm study showed that intravitreal aflibercept significantly improved the visual and anatomical outcomes of PCV that manifested with exudation from the BVN after pretreatment with PDT and/or anti-VEGF other than aflibercept. After 1 year of treatment (aflibercept injected eight times), 87.5% of patients had an improved BCVA or maintained their BCVA from baseline (35 of 40 eyes). Previous studies have reported that intravitreal injection of anti-VEGF agents, such as bevacizumab and ranibizumab, reduced exudation in eyes with PCV with various visual outcomes^11–19^. Saito *et al*.^20^ reported the efficacy of intravitreal ranibizumab injections in eyes with persisting polypoidal lesions with recurrent or residual leakage from the BVN after PDT. Eyes showed complete resolution of polypoidal lesions and improvement in BCVA and CMT after intravitreal ranibizumab injection. However, this study had a relatively small sample size and short-term follow-up period. In addition, treatment was based on an as-needed regimen. Proactive treatment, such as fixed dosing or a treat-and-extend regimen, has a longer-term effect than PRN treatment^21–24^. In the current study, we employed a fixed dosing regimen that was comparable to that used in the VIEW clinical trial to determine the effect of aflibercept in cases of PCV with exudation but without active polyps.

One study that compared the efficacy of ranibizumab and aflibercept for PCV reported that although visual outcomes did not differ significantly between the agents, aflibercept more often led to polyp regression than ranibizumab^25^. Other studies have reported that switching therapy from a different anti-VEGF agent to aflibercept was also effective at treating PCV^26,27^. Aflibercept might be effective in cases with ranibizumab tachyphylaxis because aflibercept binds all isomers of the VEGF-A and VEGF-B family as well as placental growth factor, and its binding affinity to VEGF is higher than that of ranibizumab^26,27^.

In this study, the inactivity of polyps after previous treatment was defined by the absence of polyp(s) on ICGA, or if noted, by the absence of subretinal fluid around the polyp(s) on OCT. In a recent randomized controlled trial that compared ranibizumab monotherapy with the combination therapy of ranibizumab and PDT, polyp inactivation was noted in 50% and 79% of the monotherapy and combination therapy arms, respectively^28^. In another multicenter randomized controlled trial comparing aflibercept monotherapy with the combination therapy of aflibercept and deferred PDT, the percentages of patients with no active polyp(s) at month 12 were 82% and 89% in the monotherapy and combination therapy groups, respectively^29^. These results suggest that polyp regression occurs rather frequently after intravitreal anti-VEGF treatment. Whether polyp regression prevents any future recurrence is not known. Furthermore, complete regression or inactivation of polyps as a treatment endpoint for PCV has not yet been established. Although PDT may lead to polyp regression and improvement in vision, in many cases the visual gain is not maintained over the long-term^30^. The persistence of a branching vascular network and chronic exudation related to it may lead to worse visual outcomes in recurrent cases.

We also investigated prognostic factors of treatment for active PCV without polyps. Patients with a smaller BVN and better vision at baseline and visit 4 (one month after three monthly injections) had better visual outcomes. Previous studies also reported that smaller lesion size at baseline and no recurrence during follow-up were related to a favorable visual prognosis in eyes with PCV^16,31^. It is interesting that the presence of an inner retinal cyst at visit 4 was related to worse visual outcomes. In a previous study of eyes with PCV as well as neovascular age-related macular degeneration, eyes with inner nuclear layer cystoid spaces showed worse visual outcomes^32^. The fluid in the retina seen on OCT might be a degenerative cystic change rather than exudative fluid. That is, fluid in the cystoid space in the inner retina may not be the result of a leakage from a vascular abnormality but rather the result of necrotic cell damage, which may explain the poor response to anti-VEGF treatment in those eyes.

Our results suggest that better outcomes at 14 months can be predicted by better vision, a smaller BVN, and absence of an inner retinal cyst one month after three monthly injections. This might be interpreted to mean that the findings at one month after three monthly injections will reflect the findings at 14 months after continuous bimonthly injections to some degree.

Our study had several limitations. No control group was included. Patients were treated with various treatment modalities before enrolling in the study. Because only one treatment regimen was used, the potential value of other regimens, such as combined aflibercept and PDT, was not examined. Furthermore, considering that subretinal fluid remained in 37.5% of patients despite consecutive injections every 2 months until 14 months, additional studies on more effective regimens including treat-and-extend would be worthwhile. Despite these limitations, however, we documented the efficacy of intravitreal aflibercept injections in patients with exudative PCV without active polyps who had a history of previous treatment for PCV.

In conclusion, intravitreal aflibercept improved the visual and anatomical outcomes of PCV that manifested as exudation from the BVN after pretreatment with PDT and/or anti-VEGF other than aflibercept. BCVA and the size of the BVN at baseline were significantly associated with final BCVA. Current study suggests that findings at one month after three initial monthly injections can potentially predict long-term visual outcomes.

## Methods

### Study design

This prospective, open-label, multicenter, investigator-initiated clinical trial enrolled 40 patients at Samsung Medical Center, Seoul National University Bundang Hospital and Kim’s Eye Hospital, between March 1, 2014, and September 31, 2016 (protocol in Supplement 1). Institutional Review Board approval was obtained from each participating hospital, all experimental procedures adhered to the tenets of the Declaration of Helsinki, and all subjects provided written informed consent to participate in the study. This trial was pre-registered at clinicaltrial.gov under identifier NCT02072408 (registered February 10, 2014). Inclusion criteria were as follows: (1) patients older than 20 years, (2) BCVA letter score of 24 to 77 using ETDRS charts at a starting distance of 4 m, (3) diagnosis of PCV, (4) prior treatment history of PDT and/or anti-VEGF other than aflibercept, (5) persistence or recurrence of subretinal fluid and/or inner retinal cyst on SD-OCT image after the prior treatment, (6) no active polyp(s) after the prior treatment. Thus, for those included, no polyps were noted on ICGA after prior treatment, or if noted, there was no evidence of exudation from the polyp(s) such as fluid around the polyp(s) on the OCT scan. Only one eye from each patient was included. Key exclusion criteria were as follows: (1) any concurrent progressive retinal disease, (2) history of previous vitreoretinal surgery and/or scleral buckling, (3) treatment history of laser therapy (including PDT) within 3 months, (4) treatment history of intravitreal or sub-tenon injection of triamcinolone acetonide within 3 months, (5) any history of intravitreal injection of aflibercept, (6) treatment history of intravitreal injection of ranibizumab or bevacizumab within 1 month in the study eye or fellow eye, and/or (7) history of cataract surgery within 3 months.

### Treatment administration and assessments

The treatment regimen used was similar to one of the less-intensive aflibercept regimens used in a previous clinical trial^33^. That is, patients were initially given three consecutive intravitreal injections of 2 mg aflibercept at 4-week intervals, followed by injections every 2 months in the maintenance phase.

**Figure 4:**
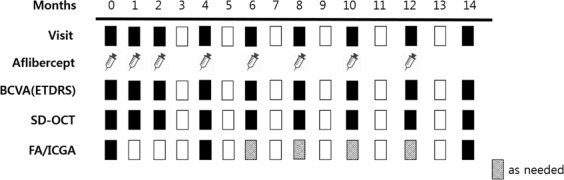
Study schedule. Patients received intravitreal injections of aflibercept (2.0 mg) every 2 months after three initial monthly doses. BCVA = best-corrected visual acuity; ETDRS = Early Treatment Diabetic Retinopathy Study; OCT = optical coherence tomography; FA = fluorescein angiography; ICGA = indocyanine green angiography.

Study schedule is shown in Fig. 4. Patients were examined every study visit. All patients underwent examination including BCVA letter score using ETDRS charts at a starting distance of 4 m, slit lamp biomicroscopy with a dilated fundus examination, fundus photography, and SD-OCT (Spectralis® HRA + OCT, Heidelberg Engineering Inc.). Fluorescein angiography and ICGA were performed at baseline and at months 4 and 14. ICGA was performed to evaluate the number of polyps and punctate hyperfluorescence spots, the BVN area, and the LGH area. Presence of subretinal fluid, inner retinal cyst, pigment epithelium detachment and subretinal hyperreflective materials was evaluated with SD-OCT. CMT was also evaluated with SD-OCT. LGH was defined as a hyperfluorescent lesion with a clearly demarcated geographic margin that became apparent approximately 10 min after the injection of ICG dye^34^. Subretinal hyperreflective material was defined as hyperreflective material located external to the retina and internal to the retinal pigment epithelium seen on OCT^35^. Size of the BVN was measured using early-phase ICGA images. Number of punctate hyperfluorescence spots and size of the LGH were assessed using late-phase ICGA images. The size of the BVN and LGH was assessed using the public-domain software ImageJ (Wayne Rasband, National Institutes of Health, USA; available at http://imagej.nih.gov/ij).

If a patient met all rescue-treatment criteria after the three initial monthly injections, the patient received PDT with verteporfin. Rescue-treatment criteria included loss of five BCVA letters, presence of subretinal fluid or an inner retinal cyst on the SD-OCT image, and the presence of an active polyp on ICGA.

### Statistical analyses

All statistical analyses were performed with the SPSS software (Version 24.0, IBM Corp, Armonk, New York, USA). The Wilcoxon signed rank test was used to compare the mean BCVA or CMT from baseline. Continuous parameters were analyzed using a generalized linear model to determine the relationships between parameters and outcomes. Logistic regression was used to compare the non- continuous parameters with other parameters. A *P* value less than 0.05 was considered to be statistically significant.

## Supplementary information

Dataset 1

## Data Availability

The datasets generated during and/or analyzed during the current study are available from the corresponding author on reasonable request.
