# Supplementary material for: Intravitreal aflibercept for active polypoidal choroidal vasculopathy without active polyps
Source: Sci Rep. 2019 Feb 6;9:1487. doi: 10.1038/s41598-018-37523-5 (PMC6365522; doi:10.1038/s41598-018-37523-5)
Supplement: Supplementary file 1 — Dataset 1 [file 41598_2018_37523_MOESM1_ESM.docx]

**Intravitreal aflibercept for active polypoidal choroidal vasculopathy without active polyps**

1. **Project summary**

The purpose of this study was to evaluate the efficacy of intravitreal aflibercept for active polypoidal choroidal vasculopathy (PCV) without active polyps and to identify prognostic factors. We enrolled patients who manifested PCV with exudation but without active polyps after prior treatment with photodynamic therapy (PDT) and/or anti-vascular endothelial growth factor (VEGF) other than aflibercept. Participants were initially given three consecutive intravitreal injections of aflibercept at 1-month intervals, followed by injections every 2 months in the maintenance phase. Spectral-domain optical coherence tomographic and indocyanine green angiographic features were assessed to determine associations between anatomical parameters and visual outcomes 14 months later. Intravitreal aflibercept improved the visual and anatomical outcomes of PCV with exudation from BVN after pre-treatment with PDT and/or anti-VEGF other than aflibercept. Better vision, smaller lesion size, and absence of an inner retinal cyst after induction therapy may predict better visual outcome.

1. **General information**
   1. Protocol title: Efficacy and Safety of Intravitreal Vascular Endothelial Growth Factor Trap-eye in Patients With Polyploidal Choroidal Vasculopathy
   2. Study Start: February 2014
   3. Clinical Trial ID: NCT02072408
   4. Sponsor: Bayer Korea

- Address: 23, Boramae-ro 5-gil, Dongjak-gu, Seoul, Republic of Korea
- Tel: +82-2-829-6600
  1. Investigator: Se Woong Kang, MD, PhD

- Address: Department of Ophthalmology, Samsung Medical Center, Sungkyunkwan University School of Medicine, #81 Irwon-ro, Gangnam-gu, Seoul, 06351, South Korea

- Tel: +82-2-3410-3562

1. **Rationale & background information**

Polypoidal choroidal vasculopathy (PCV) is characterized by the occurrence of polypoidal lesions or “polyps” that frequently progress to exudative and hemorrhagic complications in the subretinal or sub-pigment epithelial space. The etiology of PCV is unclear; it is not yet known whether it is a focal morphological manifestation of choroidal neovascularization or an abnormality of the choroid itself.

Anti–vascular endothelial growth factor (VEGF) therapy and photodynamic therapy (PDT) with verteporfin are the current mainstays of treatment for PCV. The primary mechanism of action of PDT is eliminating polyps. Thus, PDT is generally performed when active polyps are noted on ICGA. In contrast, the role of anti-VEGF is to resolve exudation by reducing the activity of the entire neovascular lesion, including the branching vascular network (BVN) as well as polyps.

Aflibercept (Eylea, Regeneron, Tarrytown, NY) is a soluble decoy receptor fusion protein that binds to all isoforms of VEGF-A and VEGF-B as well as placental growth factor. Intravitreal aflibercept injections in treatment-naive eyes with PCV improved visual acuity and macular morphology. In one recent randomized trial, aflibercept monotherapy without PDT resulted in comparable visual outcomes to combination therapy with aflibercept and deferred PDT.

Many patients have recurrent or refractory exudation but no active polyps because of previous treatment. Thus, how to manage cases of PCV with exudation but without polyps is of real-world importance. However, the efficacy of aflibercept in recurrent or refractory cases has not been determined. Furthermore, it is not clear if anti-VEGF therapy is effective in cases without active polyps.

Our aims in this study were therefore to evaluate the efficacy of intravitreal aflibercept for PCV with exudation but without active polyps in eyes pre-treated with PDT or anti-VEGF other than aflibercept, and to identify factors related to visual prognosis.

1. **Study goals and objectives**
   1. Study goal: To evaluate the efficacy of intravitreal aflibercept for PCV with exudation but without active polyps in eyes pre-treated with PDT or anti-VEGF other than aflibercept
   2. Primary Outcome Measure:
2. Change of best-corrected visual acuity (ETDRS letters)
   1. Secondary Outcome Measures:
3. Presence of subretinal hemorrhage:

Total number of months when subretinal hemorrhage are detected on fundus photographs.

1. Presence of fluid in macula evidenced by optical coherence tomography:

Total number of months when fluid in macula are detected on optical coherence tomography

1. Change of indocyanine green angiography:

Change of indocyanine green angiography will include following

a. New appearance of polypoidal structure

b. Any change in size of the greatest linear dimension of lesion

c. Any change in size and activity of branching vascular network

1. Number of eyes which need rescue treatment (photodynamic therapy):

In this study, photodynamic therapy will be performed as a rescue therapy according to the necessary criteria.

1. safety outcomes:

Frequency and severity of ocular adverse event

1. **Study Design**
   1. Prospective, open-label, multicenter, investigator-initiated clinical trial
   2. Enrolled 40 patients at Samsung Medical Center, Seoul National University Bundang Hospital and Kim’s Eye Hospital, between March 1, 2014, and September 31, 2016
   3. Inclusion criteria were as follows: (1) patients older than 20 years, (2) BCVA letter score of 24 to 77 using ETDRS charts at a starting distance of 4 m, (3) diagnosis of PCV, (4) prior treatment history of PDT and/or anti-VEGF other than aflibercept, (5) persistence or recurrence of subretinal fluid and/or inner retinal cyst on SD-OCT image, after the prior treatment, (6) no active polyp(s) after the prior treatment. Thus, for those included, no polyps were noted on ICGA after prior treatment, or if noted, there was no evidence of exudation from the polyp(s) such as fluid around the polyp(s) on the OCT scan. Only one eye from each patient was included.
   4. Key exclusion criteria were as follows: (1) any concurrent progressive retinal disease, (2) history of previous vitreoretinal surgery and/or scleral buckling, (3) treatment history of laser therapy (including PDT) within 3 months, (4) treatment history of intravitreal or sub-tenon injection of triamcinolone acetonide within 3 months, (5) any history of intravitreal injection of aflibercept, (6) treatment history of intravitreal injection of ranibizumab or bevacizumab within 1 month in the study eye or fellow eye, and/or (7) history of cataract surgery within 3 months.
2. **Methodology**
   1. The treatment regimen: patients were initially given three consecutive intravitreal injections of 2 mg aflibercept at 4-week intervals, followed by injections every 2 months in the maintenance phase.
   2. Study schedule and Follow-Up


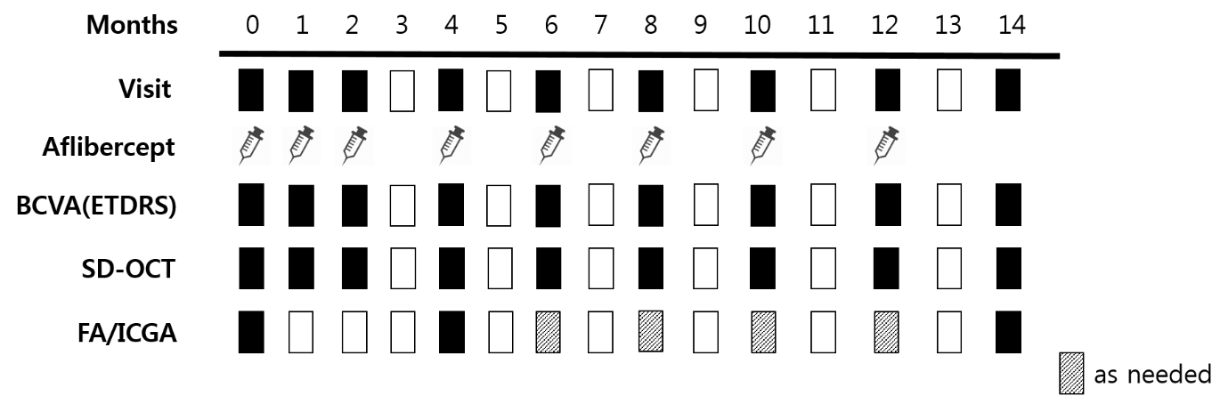


- 1. Patients were examined every study visit.

1. BCVA letter score using ETDRS charts at a starting distance of 4 m
2. Slit lamp biomicroscopy with a dilated fundus examination
3. Fundus photography, and SD-OCT (Spectralis® HRA+OCT, Heidelberg Engineering Inc.).
4. Fluorescein angiography and ICGA were performed at baseline and at months 4 and 14.
   1. If a patient met all rescue-treatment criteria after the three initial monthly injections, the patient received PDT with verteporfin.
5. Rescue-treatment criteria included loss of five BCVA letters, presence of subretinal fluid or an inner retinal cyst on the SD-OCT image, and the presence of an active polyp on ICGA.
6. **Safety Considerations**
   1. Definition of Adverse Reactions

An adverse reaction is an undesirable sign, symptom or medical condition that occurs or worsens after administration of a test drug, although it is not related to the test drug. The medical condition / disease present prior to the administration of the test drug is considered an adverse reaction only if it deteriorates after administration of the test drug. Abnormal clinical laboratory values ​​or test results may only result in adverse events if they cause clinical signs or symptoms, are clinically significant, or require treatment.

The occurrence of an adverse reaction should be sought through an indirect question when the patient visits the laboratory during the course of the study. They can also be spoken spontaneously during visits or during visits, through physical examinations, clinical laboratory tests or other assessments. All adverse events should be recorded in the 'Adverse Events Case Record' with the following information:

1. Severity rating (mild, moderate, severe)
2. Causal relationship with test drug (suspected/unsuspected)
3. Duration (start and end date or last evaluation)
4. Severe adverse event

7-2 Evaluation of adverse reactions

The researcher records information about the adverse reaction by appropriate specific questions and tests when he / she meets the subject. All adverse events that occurred during the study period were not relevant to the test drug and are documented in detail in the case record with respect to its symptoms and signs, date / end date, severity, treatment and progress, and causality with the test drug. In the event of a serious adverse reaction, follow the prompt reporting procedure and complete a critical adverse event reporting form. Patients who develop an adverse event are tracked to recovery, stabilization, or until they are concluded to be irrelevant to the test drug.

7-3 Reporting method

To ensure the safety of the subject, all significant adverse events that occurred until 30 days after the subject's participation in the clinical trial (defined as the time of final administration of the test drug or later in the final visit). Regardless of the causal relationship, immediately notify the responsible investigator immediately when the occurrence is recognized. Serious adverse events occurring after 30 days should be reported to the responsible investigator only if the tester determines that the causal relationship with the test drug is suspected. Recurrence of symptoms, worsening of complications or the first serious adverse event is also reported as a follow-up of the original symptoms as soon as the tester receives the follow-up information. If they occur at different time intervals, or if they are deemed to have nothing to do with the previously reported events, report them as new events.

All serious adverse event related information is collected and recorded in the ‘Serious Adverse Event Report Form’. The examiner must evaluate and record the causal relationship, complete the report form in English, sign it and send it immediately to the responsible investigator. The responsible investigator will, within 15 days from the day the tester is notified in accordance with the agreed procedure, To the ‘Bayer Safety Desk’ in Korea.

7-4 The management and preparation of the VEGF Trap-eye is carried out by the management pharmacist at the clinical trial center.

1. **Data Management and Statistical Analysis**

8-1 Data recording

It is the expectation of the sponsor that all data entered into the CRF has source documentation available at the site. A source document checklist will be used at the site to identify the source data for all data points collected and the monitor will work with the site to complete this.

8-2 Monitoring

In accordance with applicable regulations, GCP, and sponsor’s/CRO’s procedures, monitors will contact the site prior to the start of the study to review with the site staff the protocol, study requirements, and their responsibilities to satisfy regulatory, ethical, and sponsor’s requirements. When reviewing data collection procedures, the discussion will also include identification and documentation of source data items. The sponsor/designee will monitor the site activity to verify that the:

1. Data are authentic, accurate and complete
2. Safety and rights of subjects are being protected
3. Study is conducted in accordance with the currently approved protocol (including study treatment being used in accordance with the protocol)
4. Any other study agreements, GCP, and all applicable regulatory requirements are met.

The investigator and the head of the medical institution (where applicable) agrees to allow the monitor direct access to all relevant documents.

8-3 Data processing

Subject data necessary for analysis and reporting will be entered or transmitted into a validated database or data system. Clinical data management will be performed in accordance with applicable sponsor’s standards and data cleaning procedures. This is applicable for data recorded on eCRF as well as for data from other sources.

8-4 Statistical Analysis

All statistical analyses were performed with the SPSS software (Version 24.0, IBM Corp, Armonk, New York, USA). The Wilcoxon signed rank test was used to compare the mean BCVA or CMT from baseline. Continuous parameters were analyzed using a generalized linear model to determine the relationships between parameters and outcomes. Logistic regression was used to compare the non- continuous parameters with other parameters. A P value less than 0.05 was considered to be statistically significant.

1. **Quality Assurance**

To ensure compliance with GCP and regulatory requirements, a member of the sponsor’s (or a designated CRO’s) quality assurance unit may arrange to conduct an audit to assess the performance of the study at the study site and of the study documents originating there. The investigator/institution will be informed of the audit outcome. In addition, inspections by regulatory health authority representatives and IEC(s)/IRB(s) are possible. The investigator should notify the sponsor immediately of any such inspection. The investigator/institution agrees to allow the auditor or inspector direct access to all relevant documents and allocate his/her time and the time of his/her staff to the auditor/inspector to discuss findings and any issues. Audits and inspections may occur at any time during or after completion of the study.

1. **Expected Outcomes of the Study**

The study will contribute to discover the efficacy of intravitreal aflibercept for PCV with exudation but without active polyps in eyes pre-treated with PDT or anti-VEGF other than aflibercept. It will help to set the direction of clinical treatment.

1. **Dissemination of Results and Publication Policy**

The sponsor is interested in the publication of the results of every study it performs. All relevant aspects regarding publication will be part of the contract between the sponsor and the investigator/institution.

1. **Duration of the Project**

March 1, 2014, and September 31, 2016

1. **Problems Anticipated**

There was no control group. Patients were treated with various treatment modalities before enrolling in the study. Because only one treatment regimen was used, the potential value of other regimens, such as combined aflibercept and PDT, was not examined. Despite these limitations, however, we documented the efficacy of intravitreal aflibercept injections in patients with exudative PCV without active polyps who had a history of previous treatment for PCV.

1. **Project Management**
2. Kang S.W. (study design, data collection, data analysis, review of the manuscript, decision to submit),
3. Park K.H., Lee D.W., Kim J.H. (study design, data collection, review of the manuscript),
4. Bae K.H., Jang J.W., Lee S.E. (data analysis, writing of manuscript)
5. **Ethics**

Institutional Review Board approval was obtained from each participating hospital, all experimental procedures adhered to the tenets of the Declaration of Helsinki, and all subjects provided written informed consent to participate in the study. This trial was pre-registered at clinicaltrial.gov under identifier NCT02072408 (registered February 10, 2014).

1. **Funding/Support**

Funding was provided by Bayer for the overall study and medical writing and editorial assistance for this article.

1. **Informed Consent Forms:** attached separately at the end
2. **References**

1 Uyama, M. et al. Polypoidal choroidal vasculopathy: natural history. Am J Ophthalmol 133, 639-648 (2002).

2 Chung, S. E., Kang, S. W., Lee, J. H. & Kim, Y. T. Choroidal thickness in polypoidal choroidal vasculopathy and exudative age-related macular degeneration. Ophthalmology 118, 840-845, doi:10.1016/j.ophtha.2010.09.012 (2011).

3 Cheung, C. M. G. et al. Polypoidal Choroidal Vasculopathy: Definition, Pathogenesis, Diagnosis, and Management. Ophthalmology 125, 708-724, doi:10.1016/j.ophtha.2017.11.019 (2018).

4 Koh, A. et al. EVEREST study: efficacy and safety of verteporfin photodynamic therapy in combination with ranibizumab or alone versus ranibizumab monotherapy in patients with symptomatic macular polypoidal choroidal vasculopathy. Retina (Philadelphia, Pa.) 32, 1453-1464, doi:10.1097/IAE.0b013e31824f91e8 (2012).

5 Browning, D. J., Kaiser, P. K., Rosenfeld, P. J. & Stewart, M. W. Aflibercept for age-related macular degeneration: a game-changer or quiet addition? Am J Ophthalmol 154, 222-226, doi:10.1016/j.ajo.2012.04.020 (2012).

6 Stewart, M. W. Aflibercept (VEGF Trap-eye): the newest anti-VEGF drug. Br J Ophthalmol 96, 1157-1158, doi:10.1136/bjophthalmol-2011-300654 (2012).

7 Yamamoto, A. et al. One-Year Results of Intravitreal Aflibercept for Polypoidal Choroidal Vasculopathy. Ophthalmology 122, 1866-1872, doi:10.1016/j.ophtha.2015.05.024 (2015).

8 Iida, T. Results of the PLANET study. Paper presented at: Asia-Pacific Vitreo-retina Society Annual Meeting, Bangkok, Thailand doi:10.1097/iae.0000000000000499 (December 9, 2016).

9 Saito, M., Iida, T., Kano, M. & Itagaki, K. Two-year results of intravitreal ranibizumab for polypoidal choroidal vasculopathy with recurrent or residual exudation. Eye (Lond) 27, 931-939, doi:10.1038/eye.2013.114 (2013).

10 Lai, T. Y., Lee, G. K., Luk, F. O. & Lam, D. S. Intravitreal ranibizumab with or without photodynamic therapy for the treatment of symptomatic polypoidal choroidal vasculopathy. Retina (Philadelphia, Pa.) 31, 1581-1588, doi:10.1097/IAE.0b013e31820d3f3f (2011).

11 Lai, T. Y., Chan, W. M., Liu, D. T., Luk, F. O. & Lam, D. S. Intravitreal bevacizumab (Avastin) with or without photodynamic therapy for the treatment of polypoidal choroidal vasculopathy. Br J Ophthalmol 92, 661-666, doi:10.1136/bjo.2007.135103 (2008).

12 Kokame, G. T., Yeung, L. & Lai, J. C. Continuous anti-VEGF treatment with ranibizumab for polypoidal choroidal vasculopathy: 6-month results. Br J Ophthalmol 94, 297-301, doi:10.1136/bjo.2008.150029 (2010).

13 Cheng, C. K., Peng, C. H., Chang, C. K., Hu, C. C. & Chen, L. J. One-year outcomes of intravitreal bevacizumab (avastin) therapy for polypoidal choroidal vasculopathy. Retina (Philadelphia, Pa.) 31, 846-856, doi:10.1097/IAE.0b013e3181f84fdf (2011).

14 Oishi, A. et al. Comparison of the effect of ranibizumab and verteporfin for polypoidal choroidal vasculopathy: 12-month LAPTOP study results. Am J Ophthalmol 156, 644-651, doi:10.1016/j.ajo.2013.05.024 (2013).

15 Kang, H. M. & Koh, H. J. Long-term visual outcome and prognostic factors after intravitreal ranibizumab injections for polypoidal choroidal vasculopathy. Am J Ophthalmol 156, 652-660, doi:10.1016/j.ajo.2013.05.038 (2013).

16 Kokame, G. T., Yeung, L., Teramoto, K., Lai, J. C. & Wee, R. Polypoidal choroidal vasculopathy exudation and hemorrhage: results of monthly ranibizumab therapy at one year. Ophthalmologica 231, 94-102, doi:10.1159/000354072 (2014).

17 Inoue, M., Arakawa, A., Yamane, S. & Kadonosono, K. Long-term outcome of intravitreal ranibizumab treatment, compared with photodynamic therapy, in patients with polypoidal choroidal vasculopathy. Eye (Lond) 27, 1013-1020; quiz 1021, doi:10.1038/eye.2013.179 (2013).

18 Hikichi, T. et al. One-year results of three monthly ranibizumab injections and as-needed reinjections for polypoidal choroidal vasculopathy in Japanese patients. Am J Ophthalmol 154, 117-124 e111, doi:10.1016/j.ajo.2011.12.019 (2012).

19 Saito, M., Iida, T. & Kano, M. Intravitreal ranibizumab for polypoidal choroidal vasculopathy with recurrent or residual exudation. Retina (Philadelphia, Pa.) 31, 1589-1597, doi:10.1097/IAE.0b013e31820f4b21 (2011).

20 Freund, K. B. et al. Treat-and-extend regimens with anti-VEGF agents in retinal diseases: A Literature Review and Consensus Recommendations. Retina (Philadelphia, Pa.) 35, 1489-1506, doi:10.1097/iae.0000000000000627 (2015).

21 Chin-Yee, D., Eck, T., Fowler, S., Hardi, A. & Apte, R. S. A systematic review of as needed versus treat and extend ranibizumab or bevacizumab treatment regimens for neovascular age-related macular degeneration. The British journal of ophthalmology 100, 914-917, doi:10.1136/bjophthalmol-2015-306987 (2016).

22 Hatz, K. & Prunte, C. Treat and Extend versus Pro Re Nata regimens of ranibizumab in neovascular age-related macular degeneration: a comparative 12 Month study. Acta ophthalmologica 95, e67-e72, doi:10.1111/aos.13031 (2017).

23 Inoue, M., Yamane, S., Taoka, R., Arakawa, A. & Kadonosono, K. Aflibercept for polypoidal choroidal vasculopathy: As Needed Versus Fixed Interval Dosing. Retina 36, 1527-1534, doi:10.1097/iae.0000000000000933 (2016).

24 Cho, H. J., Kim, J. W., Lee, D. W., Cho, S. W. & Kim, C. G. Intravitreal bevacizumab and ranibizumab injections for patients with polypoidal choroidal vasculopathy. Eye (Lond) 26, 426-433, doi:10.1038/eye.2011.324 (2012).

25 Miura, M., Iwasaki, T. & Goto, H. Intravitreal aflibercept for polypoidal choroidal vasculopathy after developing ranibizumab tachyphylaxis. Clin Ophthalmol 7, 1591-1595, doi:10.2147/OPTH.S50634 (2013).

26 Saito, M., Kano, M., Itagaki, K., Oguchi, Y. & Sekiryu, T. Switching to intravitreal aflibercept injection for polypoidal choroidal vasculopathy refractory to ranibizumab. Retina (Philadelphia, Pa.) 34, 2192-2201, doi:10.1097/iae.0000000000000236 (2014).

27 Koh, A. et al. Efficacy and Safety of Ranibizumab With or Without Verteporfin Photodynamic Therapy for Polypoidal Choroidal Vasculopathy: A Randomized Clinical Trial. JAMA ophthalmology 135, 1206-1213, doi:10.1001/jamaophthalmol.2017.4030 (2017).

28 Wong, C. W. et al. Three-year results of polypoidal choroidal vasculopathy treated with photodynamic therapy: Retrospective study and systematic review. Retina 35, 1577-1593, doi:10.1097/IAE.0000000000000499 (2015).

29 Tsujikawa, A. et al. Association of lesion size and visual prognosis to polypoidal choroidal vasculopathy. Am J Ophthalmol 151, 961-972 e961, doi:10.1016/j.ajo.2011.01.002 (2011).

30 Heier, J. S. et al. Intravitreal aflibercept (VEGF trap-eye) in wet age-related macular degeneration. Ophthalmology 119, 2537-2548, doi:10.1016/j.ophtha.2012.09.006 (2012).

31 Kang, S. W., Chung, S. E., Shin, W. J. & Lee, J. H. Polypoidal choroidal vasculopathy and late geographic hyperfluorescence on indocyanine green angiography. Br J Ophthalmol 93, 759-764, doi:10.1136/bjo.2008.145862 (2009).

32 Willoughby, A. S. et al. Subretinal Hyperreflective Material in the Comparison of Age-Related Macular Degeneration Treatments Trials. Ophthalmology 122, 1846-1853 e1845, doi:10.1016/j.ophtha.2015.05.042 (2015).

**<Informed Consent Forms>**

**Vascular Endothelial Growth Factor Trap-eye in Polypoidal Choroidal Vasculopathy Assessment of efficacy and stability of intravitreal injection**

When you receive your consent to participate in clinical trials and document it, you will comply with the relevant regulations and will follow the legal process based on ethical principles based on the Helsinki Declaration.

You should read this agreement carefully before deciding whether or not you will participate in the trial. It is important that you understand why this study is carried out and what it does. You can ask any questions as you read this article about this exam. Please have enough time to decide. Please ask as many questions as you need to decide whether or not to take this test.

When you have an answer to all the questions you are wondering, and you have decided that you want to participate in this exam, please sign this document to begin participating in this exam. The exam taker (or delegate of the examiner's delegate) who has described you and this document must sign this form and complete the handwritten date. Your signature means that you have been told about the test and about the risks. Your signature on this document also means that you (or a legal custodian) want to participate in this exam.

1. **Patient Comment**

**Study Outline**

Polypoidal choroidal vasculopathy is considered to be a form of age-related macular degeneration associated with morbidity and is known to be more common in Asians than in Westerners. It is characterized by a branching vascular network with radial branching in the choroid and polypoidal vascular lesions. It induces subretinal fluid, internal retinal fluid, subretinal hemorrhage and retinal pigment epithelial detachment, resulting in decreased visual acuity. Photodynamic therapy for vascular lesions and intravitreal injection of an anti-vascular endothelial growth factor into the intravitreal space are used for treatment of active vascular choroidal vasculopathy with decreased visual acuity. Vascular endothelial growth factor is known to be an important cause of decreased visual acuity by increasing the penetration and leakage of choroidal vascular lesions due to choroidal vasculopathy. The test drug, VEGF Trap-Eye, is effective in reducing the amount of vascular endothelial growth factor and is expected to be effective in the treatment of polypoidal choroidal vasculopathy.

The efficacy and safety of VEGF Trap-Eye for patients with age-related macular degeneration have been previously reported and have been approved for the treatment of mature age-related macular degeneration in the United States, Colombia and Australia and are currently on the market.

The purpose of this study was to evaluate the efficacy and safety of injecting VEGF Trap-Eye into the eye (into the vitreous cavity) in patients with polypoidal choroidal vasculopathy without active nodules.

**Test treatment**

If you participate in this test, you will receive 8 doses intravitreal injections of VEGF Trap-Eye 2 mg for 56 weeks. The first 3 doses will be given every 4 weeks and the next 5 doses will be given every 8 weeks. If your visual acuity deteriorates despite the administration of the test drug VEGF Trap-Eye, rescue-treatment for you may be considered after 16 weeks. The rescue-treatment is photodynamic therapy.

**Visit schedules**

1. Screening visit = -3 weeks ~ 0 weeks

1) Identify personal details such as age, gender, and date of birth.

2) Ophthalmologic medical history and medical history including current disease

3) Vital sign (body temperature, blood pressure and pulse)

4) Eye examination of both eyes:

- eye examination, a series of special microscopes that can come into contact with the front of your eye, and an eye test using a lens (slit lamp biomicroscope / ophthalmoscope), IOP measurement

- Optical coherence tomography, fundus photography for retinal examination

- Retinal angiography (fluorescein angiography, indocyanine green angiography)

2. Visit 1 = Day 1 (start of injection treatment)

After reviewing all the test results, test doctor will evaluate whether you are still eligible for this test. If appropriate, the following procedure will be performed.

1) Check your health status (especially about your eyes) and changes in your medication after your last visit.

2) Vital sign (body temperature, blood pressure and pulse)

3) Eye examination of both eyes before treatment:

- A visual acuity test, a series of special microscopes that can come into contact with the front of your eye, and test using a lens (slit lamp biomicroscope / ophthalmoscope)

- Optical coherence tomography, fundus photography for retinal examination

4) Treatment for test eyes:

- VEGF Trap-Eye Injection

3. Visit 2 (4th week), visit 3 (8th week)

At Visit 1, Visit 2, and Visit 3, we will visit our clinic every four weeks and will be given an intravitreal injection of VEGF Trap-Eye.

1) Check your health status (especially about your eyes) and changes in your medication after your last visit.

2) Vital sign (body temperature, blood pressure and pulse)

3) Eye examination of both eyes before treatment:

- a visual acuity test, a series of special microscopes that can come into contact with the front of your eye, and an eye test using a lens (slit lamp biomicroscope / ophthalmoscope)

- Optical coherence tomography, fundus photography for retinal examination

4) Treatment for test eyes:

- VEGF Trap-Eye Injection

4. Visit 4 = Week 16

At Visit 4, fluorescein angiography and indocyanine green angiography will be re-evaluated and an intravitreal injection of the test drug VEGF Trap-Eye will be administered. From visit 4, you will visit at intervals of 8 weeks. If necessary, you can receive rescue-treatment using photodynamic therapy at the discretion of the tester.

1) Check your health status (especially about your eyes) and changes in your medication after your last visit.

2) Vital sign (body temperature, blood pressure and pulse)

3) Eye examination of both eyes:

- eye examination, special microscope and eye examination using lens (slit lamp biomicroscope / ophthalmoscope), IOP measurement

- Optical coherence tomography, fundus photography for retinal examination

- Retinal angiography (fluorescein angiography, indocyanine green angiography)

4) Treatment for test eyes:

- VEGF Trap-Eye Injection

- Photodynamic therapy if needed

5. Visit 24 (week 24), visit 6 (week 32), visit 7 (week 40), visit 8 (week 48)

The patient underwent an intravitreal injection of the test drug VEGF Trap-Eye at 8-week intervals. When necessary, he underwent photodynamic therapy as a rescue-treatment or test for fluoroscopic angiography, indocyanine green angiography.

1) Check your health status (especially about your eyes) and changes in your medication after your last visit.

2) Vital signs (body temperature, blood pressure and pulse)

3) Eye examination of both eyes:

- eye examination, special microscope and eye examination using lens (slit lamp biomicroscope / ophthalmoscope), IOP measurement

- Optical coherence tomography, fundus photography for retinal examination

- If necessary, retinal angiography (fluorescein angiography, indocyanine green angiography)

4) Treatment for test eyes:

- VEGF Trap-Eye Injection

- Photodynamic therapy if needed

6. Visit 9 (56th week)

Upon completion of your exam, you will have a visit to the 56th week. If for any reason you stop the exam early, you will be given a visit 15 at that point and you will be given the following examinations.

1) Check your health status (especially about your eyes) and changes in your medication after your last visit.

2) Vital sign (body temperature, blood pressure and pulse)

3) Eye examination of both eyes:

- eye examination, special microscope and eye examination using lens (slit lamp biomicroscope / ophthalmoscope), IOP measurement

- Optical coherence tomography, fundus photography for retinal examination

- Retinal angiography (fluorescein angiography, indocyanine green angiography)

**Treatment procedure**

1. Test drug injection procedure

All evaluations, including test drug injections, are conducted at the hospital as an outpatient clinic. Before the injection, first, drops anesthetic eye drops to numb your eyes, eyelashes and eyelids. When you are anesthetized, you will apply the disinfectant. Next, cover your face and test eye with a clean towel or plastic sheet to create and maintain an aseptic area during the injection.

Insert a sterilized speculum or use forceps to keep your eyelids open, and your doctor will inject the test drug into your eyes. Before or after the injection, you may drop an antibiotic solution into your test eyes to reduce the chance of infection. If this state is good, you can go home. If you are concerned about your condition, you or your doctor may be asked to stay in the hospital until your condition improves.

2. Photodynamic Therapy

Light-sensitive material (Verteporfin) is injected into your vein. After a certain period of time, when it reaches the lesion site of the choroid, you instill anesthetic eye drops to numb your eyes. After contacting the eye with a lens that can enlarge the retina, it is a method to selectively treat only the lesion area by irradiating a special laser that reacts only to the substance sensitive to light. Because it has little effect on the normal area except the lesion, it can be safely used for the treatment of lesions located in the macula. After treatment, it is sensitive to light for about 48 hours, so you should wear long clothing, masks, sunglasses, etc. in your dark room to prevent skin and eyes from exposure to light.

**Risks**

1. Information on Side Effects of Drugs / Procedures

The VEGF Trap-Eye has been injected into the eyes (within the vitreous) of more than 3,500 patients who participated in clinical trials using VEGF Trap-Eye in ophthalmic patients with age-related macular degeneration. The VEGF Trap-Eye is approved for treatment of age-related macular degeneration in the United States. Therefore, the number of patients treated with this drug is constantly increasing, and many patients have received repeated doses. The intravitreal injection of VEGF Trap-Eye into the eye was well tolerated and most of the adverse events that occurred in the test were related to the intravitreal injection rather than the drug itself.

After injection of VEGF Trap-Eye, you may experience side effects. Your eyesight may not get any better after taking the test drug during your participation in this test. Your vision may get worse for a number of reasons, such as worsening of other accompanying eye diseases. Because the test drug is currently in development, there may be side effects that are not yet known. Therefore, you should tell your doctor if you have any new symptoms.

2. Risk associated with injection procedure or VEGF Trap-Eye

The following is a list of side effects reported in clinical trials for licensing products for patients with central retinal vascular occlusion and wet age-related macular degeneration. These side effects are likely to be related to the injection procedure or medication.

1. Very common side effects (may occur in more than 1 out of 10 patients)

- Hemorrhagic spots (conjunctival hemorrhage) of the eye due to small blood vessel bleeding in the outer conjunctiva of the white eye.

1. Common side effects (between 1 and 10 patients per 100 patients)

- Rupture of the retina (retinal pigment epithelium rupture)
- Detachment of the outer layer of the retina (detachment of the retinal pigment epithelium)
- Lens cloudiness (cataract)
- Corneal surface damage (corneal erosion)
- Increased intraocular pressure
- Field of view blur
- Movement of the spot in the visual field (suspended matter in the vitreous body)
- Detachment of the vitreous from the retina (gel-like substance in the eye) (vitreous detachment)
- Pain in the eyes
- Injection site pain
- A foreign body in the eye
- Increased tear secretion
- eyelid edema
- Injection site bleeding
- Blood vessels enlargement (conjunctival hyperemia) in the outer layer of the eye causing congestion.
- The cornea is annoying (corneal edema)

1. Rare side effects (may occur in 1-10 patients per 1000 patients):

- Severe infection inside the eye (endophthalmitis)
- Retinal detachment or laceration
- Systemic allergic reactions (hypersensitivity)

3. Risk associated with photodynamic therapy

There may be risks associated with photodynamic therapy. These include eye pain / discomfort, decreased vision, decreased vision of the color, macular damage, bleeding in the eye, scarring around the retina or retina (retinal / subretinal fibrosis), and inflammation. If the laser of the photodynamic therapy burns too close to the center of vision, a scotoma (temporary or permanent blind spot) may occur. After a number of years, scars may enlarge and cause a reduction in vision. Topical anesthetic eyedrops and contact lenses can be used as part of the photodynamic therapy procedure. These risks include allergic reactions, infections, and abrasions of the cornea (scratches caused by scratching the surface of the eye). Injection of light-sensitive substances (Verteporfin) may cause injection site pain, bleeding, redness, swelling, etc. These problems usually improve soon.

4. Risk associated with eye examination

Eye drops that are routinely used to enlarge the pupil for examination the retina can make you susceptible to bright light for hours. In rare cases, the IOP may rise and this may be related to nausea and blurred vision. During the hours after using these eye drops, you should avoid driving and it can be harder to read than usual. If you have to return home before the medicinal benefits of eyedrops have disappeared, relatives or agents will have to drive. During fundus photography, you may feel a little uncomfortable during a short period of time, and you can see "spots" for a few minutes due to the bright light bulb used for taking pictures. The OCT test does not cause any discomfort to you. In the case of fluorescein angiography, indocyanine green angiography, you will receive a fluorescent dye injection in the vein. Risks associated with this include injection site bleeding, bruising, nausea, and sometimes vomiting. A slight reaction such as itching, edema or redness at the injection site or around it occurs once in 100 times. More severe allergic reactions may also occur: this includes severe edema and dyspnea, which can occur in 1 in 10,000 people, and about 1 in 222,000 people develop heart attack, stroke (blood clotting in the brain) or death can happen. If you have had a previous severe allergic reaction due to fluorescein angiography or indocyanine green angiography, you will not be able to take this test.

5. Additional potential risks

In patients receiving a drug similar to VEGF Trap-Eye, the possibility of blood clot formation (arteriovenous bleeding, embolization) after intravitreal injection has been reported. Whether VEGF Trap-Eye injections increase the risk of these events is currently unknown. Because the VEGF Trap-Eye is not a naturally occurring substance in the body, there is a possibility that an antibody against the VEGF Trap-Eye will be generated in your body. It is not known what effect these antibodies will have if your body produces antibodies, but they can block the action of the VEGF Trap-Eye and stop its efficacy. If antibodies are produced in your body, you may experience an autoimmune response. It can act against the body and cause illness. The type and severity of the disease caused by this kind of reaction is unpredictable.

6. The risk of you or your spouse getting pregnant

The effect of VEGF Trap-Eye on fetal or breastfeeding is not known. Therefore, you cannot take this test if you are pregnant, plan to become pregnant within 12 weeks of the last scheduled dose of the test drug, become pregnant, or are lactating women. If you have the opportunity to become pregnant or become pregnant after taking part in this test, you must use the appropriate method of contraception during your participation in this test. Your exam doctor will discuss several different contraceptive methods and will help you choose the most appropriate contraceptive method. You must use approved contraception during the administration of the test drug and up to 12 weeks after the last test. Acceptable contraceptive methods include stable use of oral contraceptives or other contraceptive agents during more than two menstrual cycles before screening; Intrauterine contraceptive device [IUD], bilateral tubal ligation; Vasectomy; Condoms + shampoos, foaming agents or jelly or diaphragm + shampoo, foaming agent, or jelly. If you are a male participant in this test, you must use condoms unless your partner stops menstruation, or unless you are surgically infertile.

7. Unknown / unpredictable risks

There may be any unknown or rare risk associated with intravenous injection, or with VEGF Trap-Eye or photodynamic therapy. This includes the effects of the test drug when used in combination with other drugs, or an allergic reaction to the test drug. If not treated immediately, certain allergic reactions can be life-threatening. Allergic reactions can be mild (skin rash or urticaria) or severe (dyspnea or shock).

You must notify the doctor if you have any new symptoms. If you have any new information about the test medication / procedure that may affect your participation of the test, we will notify you and ask you to sign the form indicating your decision to continue participation.

**Alternative treatment of trial participation**

You are not required to participate in this study. Your testing doctor will tell you about the alternative treatment and the benefits and risks of this alternative treatment.

**Possible benefits of participating in the test**

Your vision may improve. Although there is a scientific reason that the drug administered in this trial may have beneficial effects on polypoidal choroidal vasculopathy, we do not know what benefit to you when administered clinically. The information obtained from this study may be helpful in developing better therapies for future polypoidal choroidal vasculopathy patients.

**Payment / Reward**

You will not be charged additional costs for your participation in the exam, and you will not pay for the test medication, injection or photodynamic therapy, examination or treatment required by participation in this exam. Costs incurred as a direct result of your participation in the exam, such as transportation costs, may be reimbursed. When you visit, you will receive a certain amount of transportation fee. If you stop participating during the course of the exam, you will only be paid the transportation fee until the visit. You will not be paid for your participation in this exam. Samsung Medical Center will cover the cost of medical treatment 3 times per study for the first 24 hours of emergency treatment at the Samsung Medical Center if the damage is related to the clinical trial.

**Data protection / collection Confidentiality of information and processing of personal information**

During your participation in the exam, materials (including samples) are collected. The collected data may include your personal information and the information may be used to perform the study, to organize and analyze the results, to develop and supply more safe and effective medicines, It will be used to fulfill the obligation.

- Date of birth, gender and race

Unless otherwise required by law, your personal information may be retained until the collection and use purpose is achieved. All data collected is encrypted in numerical form to keep your identity confidential. Only the staff member of the exam has the information to link your personal and encrypted numbers. This information will be kept as long as the examiner / hospital keeps the exam information. For this study, specific items of your sensitive information collected for the same purpose are as follows.

- Information about your weight and height, medications, medical history, and diseases you are taking, and your health-related information identified through various clinical tests (see information on the testing process in this article)

Unless otherwise required by law, your sensitive information may be retained until such time as the purpose of collection and use has been achieved.

You may refuse to collect and use such personal and / or sensitive information, and you have the right to discontinue your participation in this study at any time. If you do not consent to the collection and use of personal or sensitive information, you will not be able to participate in this study, but we will not be liable to you for any other disadvantages.

You have the right to request access to personal data collected about you, and if you feel that something is not accurate, you may request an correction. Once you complete the exam, you will be able to view all your records. However, this is not after the completion of your trial, but after the last patient has finished taking the test and the results of all patients have been analyzed.

**Voluntary participation in the test**

Your decision to participate in this exam is entirely voluntary. If you decide not to take the exam, you will receive alternative treatment with your ophthalmologist without informing your reasons and without any disadvantage or loss of benefit. If you decide to participate, you can change your mind and withdraw your participation at any time without any reason. If you withdraw from the exam, your treatment or legal rights will not be affected and no new information about you will be collected. However, your consent to the collection, transmission and use of your personal data created up to that point will remain in full force and will not be revoked. If you experience side effects or other discomfort, subsequent information from you may still be collected because it may affect the safety of others.

**Drop out of test**

Participation in this test and in your test may be stopped earlier than expected if, for example, it results in a scientific or safety issue, suggesting that it is safer to stop testing for the treatment of polypoidal choroidal vasculopathy. In addition, your examiners will determine that continuation of the trial is not in your best interest, especially if you experience adverse effects that may be harmful to you or if you take medication that you should not take during the trial maybe.

Also, if your examiner determines that you need additional treatment, or if you and / or your examiner do not follow the protocol in the test plan, you will be dropped from the exam. If you miss two or more consecutive test visits, you will also be dropped from the exam.

**Signing and questioning the exam**

If you decide to take the exam, you will be asked to sign and date your consent form before you take all the test procedures. You will receive a copy of this Statement and Consent Form signed and dated. You will also receive detailed contact of your exam provider. You must carry it all the time.

If you have any questions about the test, about your rights in the exam, when you have suffered from the insured or the result of the exam, please contact your doctor. It is recommended that you ask as many questions as possible to decide whether or not to take the exam.

**Contact Information**

Your exam doctors are as follows:

Name: Kang Se Woong

Position: Professor

Affiliation: Department of Ophthalmology, Samsung Medical Center

Address: 50 Ilwon-dong, Gangnam-gu, Seoul

Contact phone number: 02-3410-1249

You may be contacted if you need to know what medication you are taking on the test, on the test itself, or in an emergency. If you have any questions about your rights as a participant in a clinical trial while participating in clinical trials, you can contact us at the number listed below at Samsung Medical Center.

Subject Protection Research Ethics Personnel Tel: 02-3410-2980

I have read the patient's consent form for the above exams, have been told about the exams and have had the opportunity to ask questions. I have signed and received a copy of the patient description and consent form.

I had the opportunity to discuss with the physician / __(name)__ about the exam.

I understand that I voluntarily participate in this study, so I understand that I will sign a written agreement and will receive a copy of the agreement after I agree.

Subject: (name) (signature) (signature date)

Legal Representative (if required): (name) (signature) (signature date)

(relationship with subject)

Research Director / Co-researcher: (name) (signature) (signature date)
